# Protein enrichment of cassava residue using *Trichodermapseudokoningii* (ATCC 26801)

**DOI:** 10.1186/s13568-015-0166-8

**Published:** 2015-12-22

**Authors:** Richard Bayitse, Xiaoru Hou, Gabriel Laryea, Anne-Belinda Bjerre

**Affiliations:** Council for Scientific and Industrial Research/Institute of Industrial Research, P.O Box LG 576, Legon, Ghana; Danish Technological Institute, Gregersensvej 1, Taastrup, 2630 Denmark

**Keywords:** Fermentation, Solid State, Protein, Cassava waste

## Abstract

Solid state fermentation of cassava residue with *Trichodermapseudokoningii* was conducted for 12 days. The fermentation was carried out at temperature of 24 °C and a pH of 5.0. Urea and ammonium sulphate were used as nutrient sources and moisture content varied at 60 and 70 %. Protein content of the unfermented cassava residue was increased from 8.4 to 12.5 % when urea was used with initial moisture content of 70 % w/v. This study showed that a maximum of 48.1 % protein enrichment was achieved using urea as a source of nutrient for the growth of the fungi, whiles ammonium sulphate achieved 36.9 % protein enrichment under the same condition.

## Introduction

The use of agro-industrial residues such as coffee pulp and husk, cassava bagasse, sugarcane bagasse, sugar beet pulp, apple pomace, declassified potatoes for efficient utilisation in value-added products has gained prominence in recent years (Ashok Pandey and Soccol 2000; Soccol and Vandenberghe 2003).

Cassava is grown in Tropical Africa, South and Central America and consumed by most people in these countries. Over the years, fermentation has been carried on cassava to produce similar or different products which are mostly associated with residue products (Akinrele 1967; Onilude 1996). Cassava peels; leaves and starch residues constitute 25 % of the cassava plant which are usually discarded as wastes after harvesting and processing, with limited utilization due to low protein, high crude fibre and cyanide contents (Iyayi and Losel 2001; Onilude 1996). Among these cassava peel is noted to be the highest and is heaped in production areas with accompanying smelly odours (Ezekiel and Aworh 2013).

Cassava is one of the most important tubers cultivated in Ghana and accounts for a daily calorie intake of 30 %, is grown by almost every farming family (FAO 2014) and is cultivated in almost every region in Ghana. Large quantities are produced in the south and middle part of Ghana, which accounts for approximately 78 % of the total cassava production in Ghana. Cassava production has increased over the years from 8,966,000 MT in 2001 to 14,240,000 MT in 2011 (SRID-MoFA 2011). Currently, Nkwanta South District is the largest producer of cassava in Ghana accounting for some 712,000 MT in 2011 (Bayitse et al. 2013).

Cassava is usually processed to obtain different relatively shelf stable intermediate and final products. Most of the cassava processing in Ghana is rural based and dominated by women. A traditional method (i.e. hand peeling) and also small scale industrial processing machines are also used. In 2011, 14,240,000 MT of cassava was produced and processed, out of which generated 3,802,080 MT of cassava peels (Bayitse et al. 2013). Peels normally consist of the thin pericarp and the thicker ring. Most processes remove both the pericarp and the thicker ring along with some pulp adhered to the peels. Analysis of the chemical composition of cassava peels indicates the following: dry matter 86.5–94.5 %; organic matter 81.9–93.9 %; crude protein 4.1–6.5 %; hemicellulose and cellulose 34.4 %; and lignin 8.4 % (Kongkiattikajorn and Sornvoraweat 2011).

Cellulosic materials can be converted to protein by cellulolytic microbes thereby improving their protein contents. The application of these microbes in solid state fermentation (SSF) for protein improvement increases the application values of cellulosic materials (Gélinas and Barrette 2007; Ugwuanyi et al. 2008). The process for converting these materials to animal feed is a potential issue. Recently, the use of cellulolytic microbes to covert cellulosic materials to non-ruminants feeds was an important issue in animal husbandry (Robinson and Nigam 2003). The economic value of biotechnological production of protein-enriched products can be enhanced if the needed carbon source (glucose) could be obtained from low-cost lignocellulosic waste, using SSF (Ezekiel and Aworh 2013). Nitrogen supplementation of the raw substrate in SSF may stimulate microbial growth or improve process efficiency and can play an important role not only as nutritive compounds but also can influence pH changes during the process (Correia et al. 2007).

Lignocellulose degrading fungi are used in industrial scale, mostly for the production of cellulases, xylanases, and for biopulping. Most investigated, used and genetically improved are fungi of *Trichoderma* spp (Himmel et al. 1999). *Trichoderma* species are very efficient in producing many extracellular enzymes and are mostly used in the food and textile industries to degrade complex polysaccharides (Ezekiel and Aworh 2013). The occurrence of pathogenic *Trichoderma* strains may be restricted to species of section *Longibrachiatum*; the species in this section are *Trichoderma longibrachiatum* and *Trichoderma citrinoviride* (Kuhls et al. 1999).

Ghana produces large quantities of agricultural waste that can be converted into food, feed, energy or industrial products for domestic use. The over 2 million MT of maize stalks, 0.5 million of maize cob and 3.8 million MT of cassava peel produced per annum in Ghana are examples of conventional feed stocks which can be converted to protein rich material through SSF, which can be used to formulate animal feed especially for the poultry and fish production sectors. For instance in Ghana the poultry industry imports about 53,000 MT of feed annually which causes over US $32 million (GCNET-MOTI 2014). Increasing value-added processing of agricultural wastes in Ghana will improve the demand for agricultural waste utilisation, increase farm profitability, provide jobs, produce protein material for feed processing industries and help minimise rural urban drift.

This research is aimed at improving the protein content of cassava residue by SSF using *Trichoderma pseudokoningii* (ATCC 26801).

## Materials and methods

### Feedstock

Cassava residue was sampled from a small scale cassava processing plant from Bawjiase, Ghana. The residue is composed of cassava peel mixed with cassava trimmings. The cassava residue was soaked in water and cleaned by removing the brown outer skin, dried at 60 °C overnight, and milled (sizes of milled particles: 74 % of particles were <0.25 mm and 26 % of particles were >0.25 and <0.45 mm in diameter).

### Cassava residue composition analysis

Dry matter content (DM) of the sample was measured by weighing the samples before and after overnight drying at 105 °C in an oven. Ash content was determined by weighing before and after ashing at 550 °C for two hours in Muffle Furnace.

Protein content was measured in Eurofins Steins Laboratorium A/S (Denmark) by measuring the total nitrogen content using Dumas method. The protein content was afterwards calculated by multiplying the total nitrogen content of the sample with a factor of 6.25.

Total starch analysis was carried out using Megazyme starch assay kit based on the use of thermostable α-amylase and amyloglucosidase (McCleary et al. 1997; Megazyme International 2014). This method has been adopted by AOAC (Official Method 996.11) and AACC (Method 76.13.01).

### Microorganism

*Trichoderma pseudokoningii* (ATCC 26801) was purchased from LGC Standards.

### Inoculum preparation

The vial containing freeze dried *Trichodermapseudokoningii* (ATCC 26801) was opened and 1 ml of sterile distilled water was added and stirred to form a suspension. The suspension was transferred aseptically to 5 ml of sterile distilled water in sterile test tube. This was then incubated at room temperature (25 °C) for 2 h undisturbed. The suspension was mix well for subculture preparation.

### Subculture preparation

The malt extract agar was sterilised with 20 µL of 200 mg/L Ampicillin to kill any possible bacteria before 2 drops of inoculum was put on the malt extract agar in a petri dish, spread to cover the surface with sterile inoculation loop under aseptic condition and incubated at 24 °C for 5 days. Spores were harvested into sterile deionized water forming about 3 × 10^7^ of spore suspension per ml.

### Solid state fermentation (SSF)

30 g of cassava residue sample was weighed into 250 ml conical flasks in duplicates. 0.3 g of Urea and Ammonium Sulphate were added separately to the samples to provide different types of nutrient for the *Trichodermapseudokoningii* growth. Moisture contents of the samples were adjusted to 60 % w/v and 70 % w/v respectively with distilled water and the moisture content was determined by Moisture Analyzer (Mettler Toledo, MJ33). The pH was adjusted to 5.0–5.4 using 0.1 M HCl. The mouth of the flasks was covered with aluminium foil. The flasks containing the samples were autoclaved at 121 °C for 15 min and allowed to cool to ambient temperature. The samples were inoculated with 2 ml of spore suspension prepared from the subculture and fermented at 24 °C for 12 days. Samples were taken aseptically at 2 days intervals and dried at 80 °C for 24 h. Protein content was measured in Eurofins Steins Laboratorium A/S (Denmark) by measuring the total nitrogen content using Dumas method. The protein content was afterwards calculated by multiplying the total nitrogen content of the sample with a factor of 6.25. The data was analysed with excel.

## Results

### Biomass composition

The analysis of chemical composition of cassava residue is given in Table 1. The residual starch of the cassava waste was 47.2 %. The protein was 5.16  % and 9.3 mg/kg cyanide was measured.

**Table 1:** Chemical composition of cassava residue

Sample	DM (%)	Ash (% DM)	Starch (% DM)	Protein (% DM)	Cyanide (mg/kg)
Cassava waste	89.7 ± 0.1	6.3 ± 0.3	47.2 ± 3.2	5.16 ± 0.3	9.30 ± 0.42

Mean values with standard deviations

### Cassava residue particle sizes

The sieve analysis of the cassava residue recorded particle sizes of 74 % <0.25 mm and 26 % were >0.25 and <0.45 mm in diameter.

### Effect of initial moisture content

Two different moisture contents (60 % w/v and 70 % w/v) were used in the experiment. Among the two, 70 % w/v recorded higher protein enrichment of 12.50 % when urea was used as initial nitrogen source; 8.89 % when ammonium sulphate was used and 6.37 % when no nitrogen was used over a period of 12 days of SSF with *Trichodermapseudokoningii* (ATCC 26801) as compared to 11.82 % protein when urea was used as initial nitrogen source; 8.24 % using ammonium sulphate and 6.08 % with no nitrogen treatment at 60 % w/v moisture (Table 2).

**Table 2:** Protein content (%) of cassava waste after varying treatments

Days	Treatments					
	Urea		Ammonium Sulphate		No nutrient	
	60 % w/v moisture	70 % w/v moisture	60 % w/v moisture	70 % w/v moisture	60 % w/v moisture	70 % w/v moisture
0	8.13 ± 0.44	8.44 ± 0.00	6.52 ± 0.00	6.50 ± 0.00	5.18 ± 0.00	5.17 ± 0.00
6	9.01 ± 0.00	9.94 ± 0.44	7.05 ± 0.00	7.10 ± 0.00	5.17 ± 0.00	5.16 ± 0.00
12	11.82 ± 0.44	12.50 ± 0.44	8.24 ± 0.00	8.89 ± 0.00	6.08 ± 0.44	6.37 ± 0.00

Mean values with standard deviations

### Protein enrichment of cassava waste after SSF

**Fig. 1:**
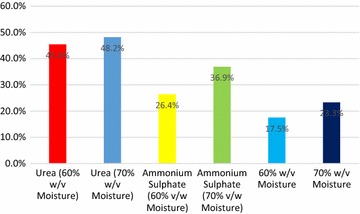
Percentage enrichment of protein content of cassava residue using different nutrient and moisture treatments

The improvement in nitrogen composition of the fermented cassava residue over 12 days is presented in Table 2. The result showed the effect of solid state fermentation with *Trichodermapseudokoningii* (ATCC 26801) on protein content of cassava residue samples. The crude protein increased from 5.17 % in unfermented samples to 6.37 % in fermented samples for cassava residue without any nutrient treatment. This accounted for only 23.34 % increase in protein enrichment over 12 days of SSF (Table 2; Fig. 1). Addition of urea and ammonium sulphate have improved protein content of the cassava residue as compared with samples without nutrient addition. Treatment with urea at 70 % moisture recorded the highest protein content of 12.50 % which showed an improvement of 48.15 % over a period of 12 days as compared to 8.89 with 36.87 % improvement over the SSF period using ammonium sulphate as nutrient source (Table 2; Fig. 1).

## Discussions

The high residual starch of cassava residue from the composition analysis could partly be attributed to the peeling process of cassava tuber which normally leaves some amount of the flesh which contains starch as well as the trimmings which are also starchy (Table 1). The total cyanide content of cassava parenchyma depends on the variety, the environment and various other factors. Cyanide levels of between 1 and 1500 ppm can be recorded (Bokanga 1994). The low cyanide content could be due to the variety of cassava (Afisiafi) as well as the pre-treatment process involving soaking in water to leach out some of the cyanide followed by drying at 60 °C for 24 h before milling. Attahdaniel et al. (2013) reduced cyanide level in cassava peel from 268 mg to 140 mg by drying. Tewe and Iyayi (1989) also reduced cyanide in cassava peel from 364.2 to 814.7 ppm to 264.3–321.5 ppm by sun drying and also confirmed that the peel of “bitter” cassava variety was shown to contain 650 ppm whiles the “sweet” variety contained an average of 200 ppm. Cyanide levels up to 1600 mg/kg have been reported in untreated cassava peel (Tivana 2012). The low cyanide content reduces the risk of fermentation inhibition which is normally associated with cassava peel as a result of high level of toxic cyanogenic glycosides (Ofoefule and Uzodinma 2009).

Particle size had a profound effect on biomass and protein production in SSF. Smaller particle sizes provide a greater saturated surface area but a lower porosity within the particles than larger particles (Camacho-Ruiz et al. 2003). Larger particles provide better aeration/respiration opportunities but provide lesser surface area which does not encourage the growth of the filamentous organism (Pandey 1992). The blend of particle sizes is promising to address the problems of aeration and surface area. An optimal particle size of 5 mm for biomass production in *Saccharomyces cerevisiae* on sugarcane baggase has been reported as well as from cassava peels with 3.35–4.00 mm using *Trichoderma viride* (Camacho-Ruiz et al. 2003; Ezekiel and Aworh 2013).

Moisture content of substrates is not entirely dependent on the environmental conditions. It is known that wood-inhabiting fungi can influence the microclimatic regime in dead wood (Tudor et al. 2012). Some fungi are able to regulate the moisture content of a substrate to ensure the optimal water availability. During the initial stages of colonization, the dry conditions are improved for optimal growth by means of cellulose and polysaccharide decomposition, while at higher levels of humidity the water surplus is extracted from the substrate into aerial mycelium, to ensure the optimal moisture content (Tudor et al. 2012). Solid state fermentation allows the manipulation of moisture in order to adjust this parameter to a particular requirements by fungal strain. The reduction in protein content with 60 % w/v moisture could be due to insufficient water which did not allow good diffusion of solutes and gas, thereby slowing cell metabolism because of a lack of substrates or through too high concentration of inhibitive metabolites in or near the cell (Gervais and Molin, 2003). High initial moisture content of more than 70 % has been reported by Ezekiel and Aworh (2013) to produce less protein in SSF using *Trichoderma viride* (ATCC 36316) which was attributed to the steric hindrance of the growth of *Trichoderma viride*. This can be caused by compaction of the substrate, reduction in porosity of the solid matrix and consequent interference with oxygen transfer.

Using *Trichoderma* species, maximum reduction of starch in cassava residue was observed during the first 12 days (Balagopalan 1996). This period is also known for optimum sugar and protein levels (Ezekiel and Aworh 2013). The range of carbon source utilised for mycelial growth of different fungi is very wide. Monosaccharides, disaccharides and polysaccharides can be used as suitable carbon sources (Nagadesi and Arya 2013). The study with *Trichodermapseudokoningii* increased protein levels in the cassava residue for all treatments which confirms its ability to secrete enzymes such as amylolytic and cellulolytic enzymes to break down starch and non-starch polysaccharides to monomer sugars which are easily metabolized to protein (Ezekiel and Aworh 2013; Balagopalan 1996). Addition of exogenous nitrogen sources increased protein enrichment during fermentation of cassava residue with *Trichodermapseudokoningii* (ATCC 26801) (Fig. 1). This agrees with the observations of Roussos et al. (1993) and Ezekiel and Aworh (2013) on the effect of nitrogen supplementation on fungal species. Urea as a nitrogen source has been utilised better by *Aspergillus oryzae* as compared with other sources (Duru and Uma 2003). Ezekiel and Aworh (2013) reported that ammonium sulphate was more effectively used by *Trichoderma viride* (ATCC 36316) than Urea.

However, in this study urea was utilized efficiently as compared to ammonium sulphate as a nitrogen source in protein production. This could be due to high nitrogen content of urea which is about 46 % as compared to 21 % in ammonium sulphate. The use of urea as initial nutrient source has increased protein enrichment by 96.2 % compared to sample without initial nutrient source. This essential element is used by fungi for functional as well as structural purposes. Chitin, the chief component of cell wall in most of the fungi, is a linear polymer of d-glucoseamine. Similarly proteins, the basis of protoplasm are composed of nitrogenous substance. Purines, pyrimidines, some vitamins and other essential metabolites are also nitrogen containing compounds. Naturally, both the organic and inorganic forms of nitrogen are available to fungi but as far as their utilization is concerned they fundamentally differ from each other in their metabolic potentialities (Nagadesi and Arya 2013).

Protein enrichment studies have been conducted on food residue such as cassava peel, pineapples and cocoyam using *Saccharomyces cerevisae* and *Aspergillus oryzae* (Duru and Uma 2003; Ezekiel and Aworh 2013; Aggelopoulos et al. 2014). Protein enrichment effect of *Trichodermapseudokoningii* on cassava residue is better as compared to *Trichoderma viride* in an experiment conducted by Ezekiel and Aworh (2013) on cassava peel. Iyayi and Losel (2001) in their work reported an increase in protein content of cassava peel from 5.6 to 14.4 % when *Aspergillus niger* was used in SSF for 20 days which was increased to 16.74 % when fermented with *Saccharomyces cerivisiea*.

In conclusion, fermentation of cassava residue with *Trichodermapseudokoningii* for 12 days could enrich the protein content by 48.2 % when urea was used as a source of nutrient and the moisture of the substrate was adjusted to 70 % w/v. About 20 % of cassava waste is only used to feed goats and sheep in Ghana because of its low protein content. From these findings, feed manufacturers will be encouraged to utilize plant proteins more efficiently to produce low-cost product for animal husbandry.
